# Supplementary material for: The interactome of intact mitochondria by cross-linking mass spectrometry provides evidence for coexisting respiratory supercomplexes
Source: Mol Cell Proteomics. 2017 Dec 8;17(2):216–32. doi: 10.1074/mcp.RA117.000470 (PMC5795388; doi:10.1074/mcp.RA117.000470)
Supplement: Supplemental Data [file supp_RA117.000470_133922_0_supp_23978_fzffwf.zip › spectra_annotation/mito_spectra_annotation/440-1-15-1-15-1.pdf]

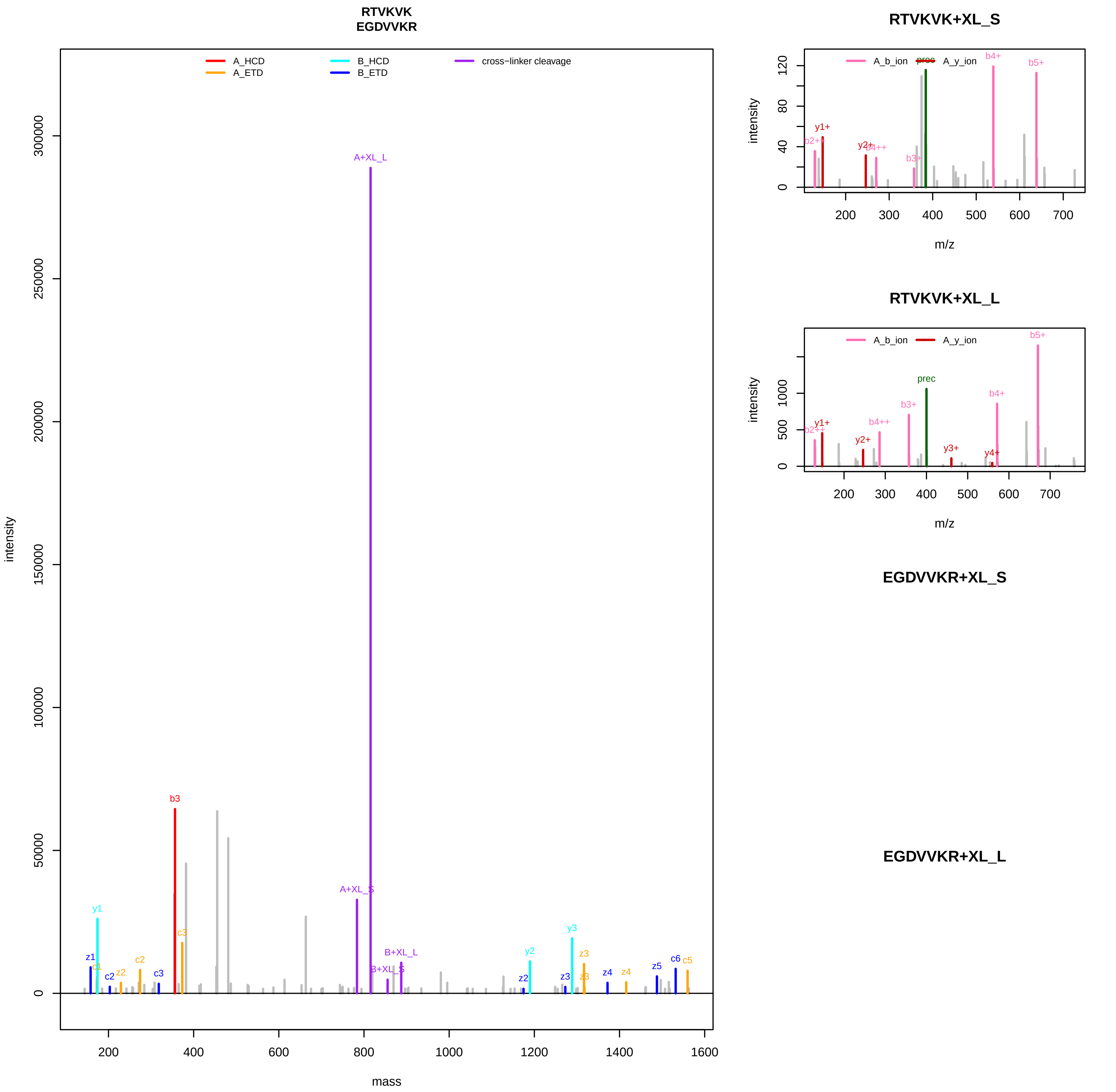

RTVKVK
EGDVVKR
A_HCD
A_ETD
B_HCD
B_ETD
cross−linker cleavage
intensity
mass
A+XL_L
A+XL_S
B+XL_L
B+XL_S
b3
y1
y2
y3
z1
c1
c2
z2
c3
z3
z4
c5
z5
c6
0
50000
100000
150000
200000
250000
300000
200
400
600
800
1000
1200
1400
1600
RTVKVK+XL_S
A_b_ion
prec
A_y_ion
b2++
y1+
y2+
b4++
b3+
b4+
b5+
m/z
RTVKVK+XL_L
y3+
y4+
EGDVVKR+XL_S
EGDVVKR+XL_L
